# Operative vs Nonoperative Management of Unstable Medial Malleolus Fractures: A Randomized Clinical Trial

**DOI:** 10.1001/jamanetworkopen.2023.51308

**Published:** 2024-01-18

**Authors:** Thomas H. Carter, William M. Oliver, Katrina R. Bell, Catriona Graham, Andrew D. Duckworth, Timothy O. White

**Affiliations:** 1 Edinburgh Orthopaedic Trauma, Royal Infirmary of Edinburgh, Edinburgh, United Kingdom; 2 Edinburgh Clinical Research Facility, Western General Hospital, Edinburgh, United Kingdom; 3 Centre for Population Health Sciences, Usher Institute, University of Edinburgh, Edinburgh, United Kingdom

## Abstract

**Importance:**

Unstable ankle fractures are routinely managed operatively. However, because of soft tissue and implant-related complications, recent literature has reported on the nonoperative management of well-reduced medial malleolus fractures after fibular stabilization, but with limited evidence supporting the routine application.

**Objective:**

To assess the superiority of internal fixation of well-reduced (displacement ≤2 mm) medial malleolus fractures compared with nonfixation after fibular stabilization.

**Design, Setting, and Participants:**

This superiority, pragmatic, parallel, prospective randomized clinical trial was conducted from October 1, 2017, to August 31, 2021. A total of 154 adult participants (≥16 years) with a closed, unstable bimalleolar or trimalleolar ankle fracture requiring surgery at an academic major trauma center in the UK were assessed. Exclusion criteria included injuries with no medial-sided fracture, open fractures, neurovascular injury, and the inability to comply with follow-up. Data analysis was performed in July 2022 and confirmed in September 2023.

**Interventions:**

Once the lateral (and where appropriate, posterior) malleolus had been fixed and satisfactory intraoperative reduction of the medial malleolus fracture was confirmed by the operating surgeon, participants were randomly allocated to fixation (n = 78) or nonfixation (n = 76) of the medial malleolus.

**Main Outcome and Measure:**

Olerud-Molander Ankle Score (OMAS) 1 year after randomization (range, 0-100 points, with 0 indicating worst possible outcome and 100 indicating best possible outcome).

**Results:**

Among 154 randomized participants (mean [SD] age, 56.5 [16.7] years; 119 [77%] female), 144 (94%) completed the trial. At 1 year, the median OMAS was 80.0 (IQR, 60.0-90.0) in the fixation group compared with 72.5 (IQR, 55.0-90.0) in the nonfixation group (*P* = .17). Complication rates were comparable. Significantly more patients in the nonfixation group developed a radiographic nonunion (20% vs 0%; *P* < .001), with 8 of 13 clinically asymptomatic; 1 patient required surgical reintervention for this. Fracture type and reduction quality appeared to influence fracture union and patient outcome.

**Conclusions and Relevance:**

In this randomized clinical trial comparing internal fixation of well-reduced medial malleolus fractures with nonfixation, after fibular stabilization, fixation was not superior according to the primary outcome. However, 1 in 5 patients developed a radiographic nonunion after nonfixation, and although the reintervention rate to manage this was low, the future implications are unknown. These results support selective nonfixation of anatomically reduced medial malleolar fractures after fibular stabilization.

**Trial Registration:**

ClinicalTrials.gov Identifier: NCT03362229

## Key Points

**Question:**

Is internal fixation of well-reduced, medial malleolus fractures superior to nonfixation after fibular stabilization when treating unstable ankle injuries?

**Findings:**

This randomized clinical trial of 154 patients found no significant difference 1 year postoperatively between fixation and nonfixation with respect to patient-reported outcomes and complications. However, 1 in 5 patients developed a radiographic nonunion after nonfixation, although the reintervention rate to manage symptoms was low.

**Meaning:**

These findings suggest that nonfixation of well-reduced medial malleolar fractures after fibular stabilization is a valid treatment option for unstable ankle fractures, particularly if the fracture is anatomically reduced.

## Teaser

This randomized clinical trial of patients with ankle fracture requiring surgery examines whether internal fixation of well-reduced medial malleolus fractures is superior to nonfixation after operative fibular stabilization.

## Introduction

Ankle fractures comprise approximately 10% of all fractures,^1^ with an annual incidence of 122 to 184 per 100 000 person-years (1:800).^2^ Conventionally, the medial malleolar component of unstable fractures has been treated through open reduction and internal fixation.^3^ Screw fixation is recommended for most, although debate continues regarding screw type, length, number, and zone of insertion.^4,5,6,7,8^ For fractures unsuitable for screw fixation, tension band wiring is recommended.^9,10,11^ High rates of symptomatic implant(s) after these techniques have driven the development of headless screws, knotless systems, and bioresorbable implants, with encouraging results.^12,13,14,15,16,17,18,19^

Previous studies have demonstrated excellent patient-reported outcomes and union rates as high as 96% in patients with isolated stable medial fractures treated conservatively.^20,21^ For unstable ankle fractures, after operative stabilization of the fibula, if the medial malleolus is well reduced, some authors have applied a similar principle of nonoperative management, reporting equivalent functional outcome when compared with fixation.^22,23,24^ This principle is based on the anatomical arrangement of the osteoligamentous complex^25^ and that consequently medial fracture fixation may not increase ankle joint stability.^24,26^ The only randomized clinical trial to date reported no statistically significant difference in functional outcome between the 2 groups at a mean follow-up of 44 months in 82 patients,^23^ with a nonoperative radiographic nonunion rate of 8%. However, this study was limited by a lack of baseline outcome data and a small sample size. The aim of the Medial Malleolus: Operative or Nonoperative (MOON) trial was to test the hypothesis that internal fixation of well-reduced medial malleolus fractures is superior to nonfixation after operative fibular stabilization in adult patients undergoing surgical management.

## Methods

This superiority-design, pragmatic (ie, under routine clinical practice conditions), prospective, parallel randomized clinical trial followed the Consolidated Standards of Reporting Trials (CONSORT) reporting guidelines.^27^ It was conducted at an academic major trauma center in the UK between October 1, 2017, and August 31, 2021, that delivers orthopedic care to a population of approximately 850 000. Data on race and ethnicity were not collected because these data were not planned for in our protocol or analysis. All participants provided informed consent. Ethical approval was granted by the South-East Scotland Research Ethics Service 2. The trial protocol has been previously published and can be found in Supplement 1.^28^

### Participants

**Figure 1.**
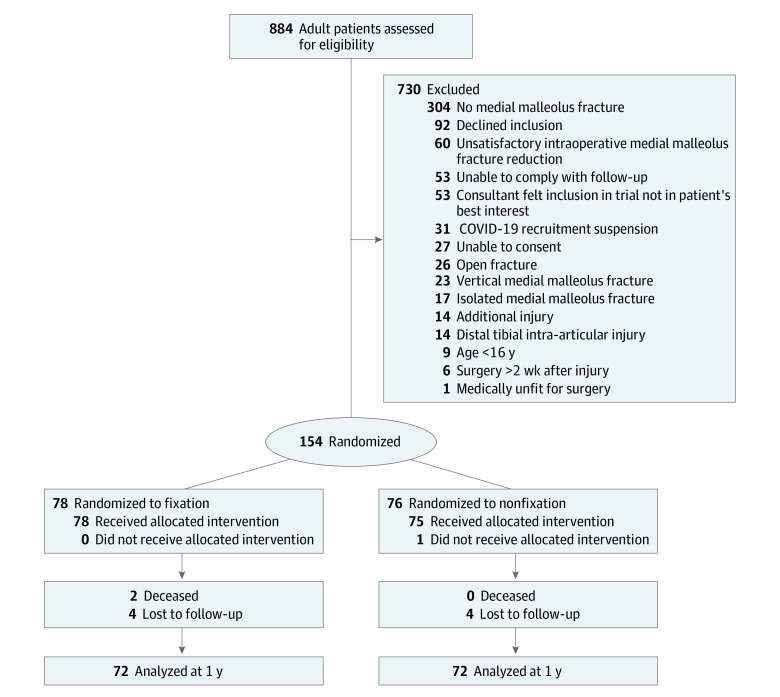
CONSORT Diagram of Trial Participant Flow

Adult patients (aged ≥16 years) presenting within 2 weeks of injury with an unstable bimalleolar or trimalleolar ankle fracture were considered. Patients were excluded if they did not have a fracture of the medial malleolus, were unable to give consent or comply with follow-up, had an open fracture, had an associated neurovascular injury, or had a significant associated lower limb injury that would affect rehabilitation. Patients with isolated medial malleolus and vertical shear fractures were excluded (Figure 1). Because of the health care impact of the SARS-CoV-2 pandemic, recruitment was suspended between February 1, 2022, and June 15, 2022. During this time, 31 potential participants were missed.

### Randomization

Randomization was allocated on a 1:1 ratio and stratified by age (<65 years and ≥65 years, which was factored into the computer-generated block design [mixed block sizes]). An independent statistician generated the randomization schedule, and individually numbered opaque envelopes were produced. As participants were enrolled in the trial, the next envelope was placed into each participant’s notes, which accompanied them to the operating theater. Once the surgeon had confirmed satisfactory medial malleolus fracture reduction, after fibular stabilization (≤2 mm displacement on anteroposterior fluoroscopy), the envelope was opened by an independent theater team member. If the medial malleolus fracture was not reduced within acceptable limits (n = 60), the envelope was not opened and was returned, in order, to the randomization sequence, and the patient was excluded.

### Interventions

Patient care was supervised by a consultant orthopedic trauma surgeon, and all surgeons, including trainees, were educated on the trial methods. Trial participants randomized intraoperatively to the fixation group were treated with open reduction and internal fixation of the medial malleolus fracture. The approach and implant type, including number and length of screws if used, were at the surgeon’s discretion. Wound closure and postoperative management, including weight-bearing prescription and immobilization, were also at their discretion. The default local postoperative protocol advised immediate full weight-bearing in a removal orthosis unless a confirmed syndesmotic injury, peripheral neuropathy, or significant associated soft tissue injury was identified. Range-of-motion exercises were permitted from 2 weeks postoperatively. Trial participants randomized intraoperatively to nonfixation had no medial fixation performed. Wound closure and postoperative management were as per the fixation group.

### Outcomes

Given the nature of the interventions, it was not practical to blind participants or outcome assessors to allocation. Outcomes were collected in person at baseline, 6 weeks, and 1 year. Postal questionnaires were completed at 3 and 6 months to minimize participant inconvenience. In some situations, it was not possible to review participants in person at the final postoperative stage because of SARS-CoV-2 pandemic hospital restrictions. In this situation, outcome scores were collected by postal questionnaire, and participants were invited for radiographic review once it was deemed safe.

The primary outcome was the Olerud-Molander Ankle Score (OMAS) at 1 year after randomization.^29^ The OMAS is validated and extensively used in ankle trauma research. It assesses outcome across 9 domains, including pain, stiffness, swelling, stair climbing, running, jumping, squatting, supports, and work or activities of daily living. A score of 0 represents the worst possible outcome and 100 represents the best possible outcome.

Secondary outcomes included the Manchester-Oxford Foot Questionnaire,^30^ with a score of 0 representing the best outcome and 100 representing the worst outcome, and the EuroQol-5D-3L (EQ-5D) standardized instrument as a measure of health outcome,^31^ with a score of +1.0 representing the best outcome or health and a score of −0.59 representing the worst. Assessment of pain, health, and satisfaction was performed using a visual analog scale, ranging from 0 to 100, with 0 representing the worse score and 100 representing the best score. In those participants employed and/or engaged in physical activity before injury, return time was recorded.

Complications were documented and classified as minor if they did not require further surgical intervention or major if they required subsequent surgery. Minor complications included superficial wound infection, wound dehiscence or delayed healing, implant prominence, localized pain, and asymptomatic nonunion. Major complications included wound infection requiring debridement, failed fixation resulting in loss of talar reduction, symptomatic nonunion, and symptomatic implants requiring removal. Medical complications, including venous thromboembolism, were documented.

Radiographic outcomes included documentation of medial malleolar fracture type according to the Herscovici classification^20^ and assessment of reduction quality based on intraoperative fluoroscopy. Reduction quality was confirmed postoperatively by reviewing intraoperative fluoroscopy and classified as anatomical, fair (≤2-mm displacement), or poor (>2-mm displacement).^21,22^ Postoperative computed tomography imaging was not performed routinely. Final radiographs were used to assess fracture union and the development of posttraumatic osteoarthritis, classified according to a modification of the Kellgren-Lawrence system, as described by Holzer et al.^32^

### Statistical Analysis

The trial was powered to detect a difference of 10 points in the OMAS 1 year after surgery between the 2 groups. At the time of power calculation, no published minimum clinically important difference (MCID) for the OMAS existed. However, after ankle fracture, a difference of 11 points had been demonstrated in patients developing posttraumatic degeneration compared with those without.^29^ Consequently, a 10-point difference was chosen. A sample size of 128 participants (64 per group) was required for 80% power, assuming an SD of 20,^33^ and 5% (2-sided) significance. The sample size was increased by 20% to account for loss to follow-up, generating a sample size of 154. Intention-to-treat analyses were performed.

The design of this trial assessed whether internal fixation of well-reduced medial malleolus fractures after satisfactory fibular stabilization is superior to nonfixation. The primary outcome was compared between groups using a 2-sample, unpaired *t* test or nonparametric equivalent, dictated by data normality. Secondary outcomes were analyzed comparably. A sensitivity analysis was performed based on the randomization stratification with an age cutoff of 65 years. Analysis of covariance was used to examine the primary outcome adjusting for baseline demographics, preinjury OMAS, and fracture classification. Comparison of binary outcomes, such as the presence of nonunion, were performed using a binominal test for comparison of proportions. Mixed-model analysis was performed by plotting the OMAS at multiple time points during recovery. Two-tailed *P* values were presented, and *P* < .05 was used to indicate statistical significance. Analysis was performed using SAS software, version 9.4 (SAS Institute Inc). Data analysis was performed in July 2022 and confirmed in September 2023.

## Results

Among 154 participants randomized (mean [SD] age, 56.5 [16.7] years; 119 female [77%] and 35 male [23%]), 144 (94%) completed the trial. Seventy-eight were allocated to fixation and 76 to nonfixation (Figure 1). Baseline demographic, injury, and clinical characteristics were similar (Table 1), including underlying comorbidities, such as diabetes, cardiac disease, kidney failure, and chronic liver disease. There was 1 crossover for a participant randomized to nonfixation, but the medial malleolus fracture was felt to redisplace when stressing the syndesmosis and therefore internally fixed. No significant differences were found between intraoperative data except for a significantly reduced tourniquet time in the nonfixation group (mean [SD] difference, −22.14 [5.70] minutes; 95% CI, −27.70 to −16.59 minutes; *P* < .001). Perioperative data are summarized in the eTable in Supplement 2.

**Table 1.** Patient Characteristics at Baseline^a^

Characteristic	Fixation (n = 78)	Nonfixation (n = 76)
Age, mean (SD) [range], y	57.1 (16) [21-87]	55.9 (17) [19-92]
Sex		
Male	13 (17)	22 (29)
Female	65 (83)	54 (71)
BMI, median (IQR)	26.2 (23.7-29.6)	28.6 (24.6-32.4)
Smoker	16 (21)	18 (24)
Alcohol intake	53 (68)	48 (63)
Engaged in employment pre-injury	42 (54)	39 (52)
Engaged in physical activity before injury	43 (55)	33 (43)
Baseline mean OMAS, mean (SD) [range]	94 (11) [55-100]	91 (15) [45-100]
Mechanism of injury		
Fall standing height	66 (85)	63 (83)
Fall from height	3 (4)	1 (1)
Direct blow	1 (1)	2 (3)
Sport	7 (9)	9 (12)
Assault	1 (1.9)	0
Lauge-Hansen classification		
Supination external rotation	71 (91)	62 (82)
Pronation external rotation	4 (5)	8 (10)
Pronation abduction	3 (4)	6 (8)
Supination adduction	0	0
AO/OTA classification		
44B2	31 (40)	29 (38)
44B3	40 (51)	35 (46)
44C2	3 (4)	4 (5)
44C3	4 (5)	8 (11)
Herscovici medial malleolus classification		
Type A	1 (1)	0
Type B	45 (58)	43 (57)
Type C	30 (38)	29 (38)
Type D	2 (3)	4 (5)
Posterior malleolus fracture present	45 (58)	47 (62)
Radiographic syndesmosis injury present	7 (9)	11 (14)

Abbreviations: AO/OTA, Arbeitsgemeinschaft für Osteosynthesefragen/Orthopaedic Trauma Association; BMI, body mass index (calculated as weight in kilograms divided by height in meters squared); OMAS, Olerud-Molander Ankle Score.

^a^ Data are presented as number (percentage) of patients unless otherwise indicated.

### Primary Outcome

**Figure 2.**
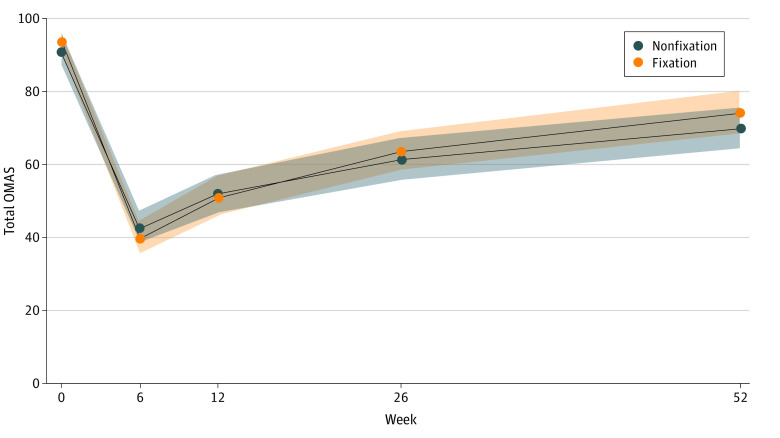
Mixed-Model Analysis Mixed-model analysis demonstrating change in mean Olerud-Molander Ankle Score (OMAS) over time by treatment group. Shaded areas indicate 95% CIs.

There were 72 trial participants analyzed in each group at the primary outcome point. Two participants in the fixation group died during the study period from unrelated causes (cardiac event and metastatic cancer). Four participants in each group could not be contacted. The median OMAS was 80.0 (IQR, 60.0-90.0) in the fixation group and 72.5 (IQR, 55.0-90.0) in the nonfixation group (Table 2). This difference of 7.5 was not statistically significant. A sensitivity analysis demonstrated no significant difference between the groups when adjusting for an age cutoff of 65 years. Analysis of covariance, adjusting for baseline OMAS, age, sex, and fracture classification, found no statistically significant difference between the 2 groups (*r*^2^ = 0.156, *P* = .42). The mean OMAS was plotted during the recovery using mixed-model analysis with treatment group and weeks as fixed effects and participant as a random effect, with no difference found between groups (Figure 2).

**Table 2.** Trial Outcome Measures (Mann-Whitney *U* Test)

Outcome measure	Median (IQR)		*P* value
	Fixation	Nonfixation	
**Preoperative**			
OMAS	100 (55-100)	100 (45-100)	.35
EQ-5D	1.00 (0.81-1.00)	1.00 (0.37-1.00)	.29
**6-8 Weeks**			
OMAS	40 (22.5-55)	40 (30-55)	.38
MOXFQ	45 (27-63)	46 (29-65)	.79
EQ-5D	0.69 (0.59-0.80)	0.66 (0.46-0.75)	.21
Health	80 (70-90)	80 (65-90)	.16
Pain	90 (70-95)	90 (70-93.5)	.92
Satisfaction	100 (90-100)	100 (99.5-100)	.23
**12 Weeks**			
OMAS	50 (35-70)	55 (35-70)	.72
MOXFQ	43 (24-59)	46 (27-64)	.50
EQ-5D	0.69 (0.62-0.80)	0.69 (0.62-0.80)	.99
Health	80 (70-90)	80 (70-90)	.56
Pain	80 (60-90)	80 (60-90)	.55
Satisfaction	90 (80-100)	90 (80-100)	.55
**26 Weeks**			
OMAS	65 (50-80)	65 (40-80)	.64
MOXFQ	28 (9-24)	34 (13-50)	.29
EQ-5D	0.76 (0.69-0.80)	0.73 (0.69-0.80)	.53
Health	90 (70-90)	80 (70-95)	.21
Pain	90 (70-95)	80 (60-90)	.12
Satisfaction	90 (90-100)	90 (90-100)	.48
**52 Weeks**			
OMAS	80 (60-90)	72.5 (55-90)	.17
MOXFQ	12 (2-32)	19 (5-41)	.16
EQ-5D	0.85 (0.71-1.00)	0.80 (0.70-0.85)	.05
Health	90 (80-95)	90 (70-95)	.23
Pain	95 (90-100)	90 (77.5-100)	.55
Satisfaction	100 (90-100)	100 (90-100)	.90
Return to work (weeks)	9 (4-12)	9 (6-12)	.60
Return to sport (weeks)	12 (10-20)	12 (8-15)	.28

Abbreviations: EQ-5D, EuroQol-5D-3L; MOXFQ, Manchester-Oxford Foot Questionnaire; OMAS, Olerud-Molander Ankle Score.

### Secondary Outcomes

With the exception of the 1-year EQ-5D score, which favored fixation (median score, 0.85 vs 0.80; *P* = .049), there was no significant difference in any of secondary outcome measures between the groups at any time point. Similarly, the proportion of patients returning to work and activity, including return time, were comparable (Table 2).

### Complications and Adverse Events

In total, 43 participants (60%) in the fixation group experienced 1 or more complications within 1 year of surgery compared with 45 (63%) in the nonfixation group (odds ratio [OR], 0.89; 95% CI, 0.46-1.74; *P* = .73) (Table 3). Four patients in the fixation group and 9 patients in the nonfixation group experienced a major complication (OR, 0.41; 95% CI, 0.12-1.40; *P* = .16). Localized medial pain was more common in the fixation group, but this finding was not statistically significant (16 patients [22%] in the fixation group vs 8 patients [11%] in the nonfixation group; OR, 2.29; 95% CI, 0.91-5.74; *P* = .08). Two patients in the fixation group required implant removal because of screw backout and irritation of the tibialis posterior tendon, respectively.

**Table 3.** Complications and Radiographic Outcomes

Outcome	No. (%) of patients	
	Fixation (n = 72)	Nonfixation (n = 72)
**Minor complications (no requirement for surgery)**		
Superficial infection		
Lateral	8 (11)	9 (13)
Medial	5 (7)	1 (1)
Wound dehiscence		
Lateral	2 (3)	2 (3)
Medial	0	0
Implant prominence		
Lateral	19 (26)	19 (26)
Medial	11 (15)	0
Localized pain		
Lateral	7 (10)	14 (19)
Medial	16 (22)	8 (11)
**Major complications (requirement for surgery)**		
Deep infection requiring surgery		
Lateral	1 (1)	2 (3)
Medial	0	0
Loss of talar reduction requiring revision	0	3 (4)
Medial malleolar nonunion requiring surgery	0	1 (1)
Removal of symptomatic implants		
Lateral	1 (1)	3 (4)
Medial	2 (3)	0
**Radiographic outcomes^a^**		
Radiographic nonunion		
Lateral	0	0
Medial	0	13 (20)
Symptomatic nonunion		
Lateral	0	0
Medial	0	5 (8)
New radiographic degeneration		
Overall	17 (25)	17 (26)
Grade 1	12 (18)	10 (15)
Grade 2	4 (6)	4 (6)
Grade 3	1 (1)	0
Grade 4	0	3 (5)

^a^ Sample sizes for radiographic outcomes are 67 for the fixation group and 65 for the nonfixation group.

### Radiographic Outcomes 

Because of the impact of the SARS-CoV-2 pandemic, radiographic data were available from 67 participants in the fixation group and 65 participants in the nonfixation group (86% in both groups) (Table 3). Some participants declined review because of ongoing concerns regarding the pandemic, in particular elderly patients and/or those with significant comorbidities. Lateral malleolar union occurred in all patients. Medial-sided union was achieved in 52 of 65 cases (80%) in the nonfixation group (eFigure 1 in Supplement 2) and all cases in the fixation group. Of the 13 patients (20%) who developed a nonunion, 8 had no localized symptoms and were classed as having asymptomatic nonunions (eFigure 2 in Supplement 2). Of the 5 participants with localized symptoms, 1 required further surgery, whereby the nonunited distal fragment was excised with improvement. All nonunions were type B fractures according to the Herscovici classification.^20^ Five nonunions occurred in 13 smokers (39%) compared with 8 nonunions in 52 nonsmokers (15%; OR, 3.44; 95% CI, 0.89-13.22; *P* = .07). We found no between-group difference in posttraumatic osteoarthritis (17 patients [25%] in the fixation group vs 17 patients [26%] in the nonfixation group; OR, 0.96; 95% CI, 0.44-2.10; *P* = .92) 1 year after surgery (Table 3).

### Effect of Medial Malleolar Nonunion and Reduction Quality

Participants with a radiographic nonunion at 1 year (n = 13) had a poorer median OMAS of 70.0 (IQR, 30.0-72.5) compared with 80.0 (IQR, 55.0-80.0) in those with radiographic union (*P* = .01). Participants with painful nonunion (n = 5) had a significantly poorer median OMAS of 20.0 (IQR, 5.0-55.0), and participants who developed an asymptomatic medial malleolar nonunion had a median OMAS of 70.0 (IQR, 67.5-80.0), which was comparable to 75.0 (IQR, 55.0-90.0) in the union group (*P* = .54). There was no difference between the type of medial malleolar fracture type (Herscovici types A-D) and the primary outcome. Intraoperative fracture reduction quality significantly affected the OMAS at 1 year (anatomical: 80.0 [IQR, 70.0-97.5], fair: 60.0 [IQR, 47.5-80.0], and poor: 60.0 [IQR, 32.5-80.0]; *P* = .008), although the study was not powered to detect a difference between fracture types or reduction quality. Participants in the nonfixation group with type C fractures that reduced anatomically (n = 17) all united (eFigure 1 in Supplement 2) and achieved satisfactory functional outcome.

## Discussion

The primary outcome of this study does not support the hypothesis that fixation of well-reduced medial malleolus fractures is superior to nonfixation in the surgical management of unstable ankle fractures. However, a 20% radiographic nonunion rate after nonfixation is notable, and although the rate of reintervention for this was low because of it being asymptomatic in most cases, the future implications are unclear. The findings support selective nonfixation of anatomically reduced fractures, with longer-term follow-up required to validate these findings.

The results of the current trial somewhat contrast with those from the study by Hoelsbrekken et al,^23^ who reported a similar OMAS in the fixation group (mean score, 80.0) but a superior, yet not statistically so, OMAS in the nonfixation group (mean score, 81.0). The aforementioned trial published mean OMAS values compared with median values in the current trial, making it difficult to draw strict comparisons. The nonunion rate in the nonfixation group was also lower than that in the current study (9% vs 20%), but the current study has likely overestimated this, as acknowledged in the Limitations section. Carter et al^22^ retrospectively compared nonfixation with fixation of medial malleolus fractures after fibular nail fixation. In that study of 54 patients treated nonoperatively, a median OMAS of 85.0 compared favorably with 80.0 in the fixation group, which included 193 patients. However, the study was limited by loss to follow-up and potential patient selection bias. In the current study, quality of life measured by the EQ-5D improved in the fixation group, particularly at 1 year; however, this finding was below the published MCID.^34^ Longer-term surveillance of quality of life will be important because deterioration may reflect chronic ankle issues, including posttraumatic degeneration.

Pinski et al^24^ reviewed 257 patients, 87 of whom had a medial malleolus fracture that was treated without fixation after fibular stabilization, and reported pain scores rather than functional outcome. In contrast to the results of the current trial, nonoperative treatment of supracollicular fractures, similar to Herscovici type C injuries, was associated with increased pain compared with fixation. We cannot explain these differences with data from the current trial, because type C fractures were successfully treated conservatively without increased risk of nonunion, pain, or poor functional outcome. We hypothesize that the larger metaphyseal fracture bed aids union and is less likely to have periosteum interposed, in contrast with smaller and more distal type B fractures. In a study of 57 conservatively managed cases, Herscovici et al^20^ found a nonunion rate of only 4% (n = 2). Both were type C fractures but were displaced by 3 and 4 mm, respectively, making it difficult to draw comparisons with the current trial and suggesting that any difference is likely due to chance.

When considering all fracture types and reduction quality as a group, the OMAS difference at 1 year was 7.5, which was not statistically significant or clinically relevant as defined by a 10-point margin in the OMAS. However, although this trial was not powered to compare individual medial fracture types and reduction quality, there was a trend highlighting comparable rates of union and functional outcome in type C fractures that remain well reduced after fibular stabilization. This finding should inform future research on this topic.

The rate of soft tissue complications after fixation was not significantly higher than with nonfixation. Localized medial-sided pain was twice as common after fixation, but this finding did not reach statistical significance. Although the results of this trial suggest that internal fixation is not required in well-reduced medial malleolar fractures, fixation was not associated with unacceptably high numbers of complications, particularly related to implant removal. Our center recommends additional surgery only for intolerable symptoms, which may explain why only 2 patients in the fixation group required implant removal. All internally fixed fractures were approached via open reduction and not percutaneously. Although percutaneous fixation is associated with a risk of delayed union,^35^ it has been shown to be safe,^36,37^ and future level 1 research comparing nonfixation with percutaneous fixation would meaningfully add to the literature. Finally, operative time in the nonfixation group was a mean of 22.14 minutes shorter. This difference could not only impart benefits for patients but also positively influence operating room flow, costs, and productivity.

### Limitations

This trial has limitations. The study was powered to detect a 10-point difference in the OMAS at 1 year. Since trial completion, MCID values for the OMAS after ankle fracture have been published, ranging from 4.3 to 11.4, influenced by timing and method of calculation after injury.^38,39,40^ A difference of 7.5 points in the current trial may therefore be viewed as clinically significant dependent on which MCID value is referenced. The results collected were from a single center and therefore may not represent general orthopedic practice, although patient care was supervised by 12 orthopedic trauma surgeons and followed a pragmatic approach. Reflecting this design, 5 participants in the nonfixation group were included in the analysis despite demonstrating poor reduction (>2-mm displacement) because the surgeon had deemed the reduction satisfactory intraoperatively. The inclusion of these patients may have influenced functional outcome and overestimated the nonunion rate. To reduce postoperative stiffness, patients were permitted to perform range-of-motion exercises out of their removable orthoses at 2 weeks after surgery. This may have increased fracture site motion in the nonfixation group, contributing to the nonunion rate. Given the nature of the interventions, blinding was not logistically possible, particularly for the primary outcome measure used. We excluded 26 patients with open fractures (4% of the excluded group), with most having a medial laceration. Considering the potential for increased soft tissue complications with these injuries, nonfixation may in fact be more beneficial to this specific cohort.^41^ Although patient outcomes, including complications, have been documented to 1 year, the implications of a clinically silent radiographic nonunion may not be recognized for several years, and longer-term surveillance is required. For participants in the fixation group, numerous surgeons saved only final intraoperative imaging, and consequently a retrospective assessment of the medial malleolar reduction for quality control purposes cannot be performed in the same manner as for the nonfixation group. Finally, some authors have identified issues with fracture malreduction when performing postoperative computed tomography imaging.^42,43^ Computed tomography was not performed in the current trial but may be considered in futures studies of this nature.

## Conclusions

In this randomized clinical trial of patients with unstable ankle fractures involving well-reduced medial malleolus fracture, internal fixation after fibular stabilization did not significantly improve functional outcomes 1 year after surgery. However, the nonunion rate and associated patient-reported outcome are important findings and could be influenced by fracture type and reduction quality, which must be scrutinized radiographically before electing to treat without fixation. Consequently, we caution against nonfixation in fractures that remain displaced after fibular stabilization. Future follow-up and monitoring for posttraumatic degeneration and its implications is recommended.

## Notes

Supplement 1.
Trial Protocol

Supplement 2. **eTable.** Perioperative Data for the MOON Trial **eFigure 1.** A Radiographic Example of a Type-C Medial Malleolar Fracture That Progressed to Satisfactory Radiographic Union **eFigure 2.** A Radiographic Example of a Type-B Medial Malleolar Fracture That Progressed to an Asymptomatic Radiographic Nonunion

Supplement 3.
Data Sharing Statement
