# Supplementary material for: Operative vs Nonoperative Management of Unstable Medial Malleolus Fractures: A Randomized Clinical Trial
Source: JAMA Netw Open. 2024 Jan 18;7(1):e2351308. doi: 10.1001/jamanetworkopen.2023.51308 (PMC10797457; doi:10.1001/jamanetworkopen.2023.51308)
Supplement: Supplement 1. — Trial Protocol [file jamanetwopen-e2351308-s001.pdf]

B

‘MOON Study’

Medial malleolus: Operative Or Non-operative

A prospective randomized controlled trial of operative versus non-operative management of associated medial malleolus fractures in unstable fracture dislocations of the ankle joint

Chief Investigator: Mr Tim O White MD, FRCSEd

Co-Investigators: Mr Tom H Carter MRCSEd

Mr Andrew D Duckworth PhD, FRCSEd

Sponsors: NHS Lothian R&D

Co-sponsors	*Scottish Orthopaedic Research Trust into Trauma (SORT-IT). Registered charity: SCO20955*
Funder	N/A
Funding Reference Number	N/A
Chief Investigator	Mr Tim White – Consultant Orthopaedic Trauma Surgeon
EudraCT Number	2017-003713-25
REC Number	17/SS/0124
ISRCTN Number	
Version Number and Date	Version 2.0, 22nd September 2017

SUMMARY

**STUDY TITLE**	Medial malleolus: Operative Or Non-operative. A prospective randomized controlled clinical trial of operative versus non-operative management of associated medial malleolus fractures in unstable fracture dislocations of the ankle joint.
**BACKGROUND**	There are limited data reporting the outcome of patients with non-operatively managed medial malleolus fractures compared to those treated surgically in the presence of lateral malleolus fixation. Conservative management would potentially result in fewer complications, reduced operative times and cost.
**STUDY OBJECTIVES**	To determine whether:<br><br>(a) PRIMARY OUTCOME: any difference exists in the patient-reported outcome measures (OMAS) at one-year after injury between operative and non-operative treatment of the medial malleolus in combination with operative fixation of the lateral malleolus<br><br>(b) SECONDARY OUTCOME: any difference in surgical complications, loss of reduction, pain, satisfaction, return to activity
**STUDY POPULATION**	Patients aged 16 years and older with isolated, closed unstable fracture dislocations of the ankle joint.
**STUDY TREAMENT**	1. Open reduction internal fixation of the medial malleolus in combination with operative fixation of the lateral malleolus.<br><br>2. Non-operative management of the medial malleolus in combination with operative fixation of the lateral malleolus.
**STUDY ASSESSMENTS**	**Primary**: Olerud-Molander Ankle Score (OMAS) at one year<br><br>**Secondary**: Complications, Manchester-Oxford Foot Questionnaire (MOXFQ), EQ-5D, Tourniquet time, Range of movement, Pain, Return to work & sport, Satisfaction, Radiographic union & early degeneration, Cost.

Table of Contents

1 BACKGROUND ... 6
1.1 Current literature ... 8
2 RESEARCH AIMS ... 10
2.1 Primary aim and null hypothesis ... 10
2.2 Secondary null hypothesis ... 10
3 PATIENTS AND METHODS ... 11
3.1 Patients and centres ... 11
3.2 Inclusion criteria ... 12
3.3 Exclusion criteria ... 13
3.4 Patient identification and consent ... 13
3.5 Interventions ... 16
4 OUTCOME AND FOLLOW-UP ASSESSMENTS ... 18
4.1 Primary outcome ... 18
4.1.1 Power analysis ... 18
4.2 Secondary outcomes ... 19
4.2.1 Statistical Analysis ... 19
4.3 Assessment Points ... 21
5 ETHICAL STATUS AND FUNDING ... 23
5.1 Ethical Status ... 23
5.2 Funding ... 23
5.3 Data Protection ... 23
5.4 Data Storage ... 24
5.5 Confidentiality ... 24
6 References ... 25

1. BACKGROUND

Ankle fractures are the second most common Orthopaedic trauma presentation, accounting for approximately 10% of the acute workload. [1] The annual incidence of ankle fractures is approximately 122-184/100,000 person years (1:800). [2] The incidence amongst elderly patients has increased over the last 30 years and with the average age expectancy constantly rising this trend will no doubt continue. [3, 4] The most basic classification system for ankle fractures, put forwards by Pervical Pott, takes into account the number of malleoli involved.[5] The lateral and medial malleoli are important contributors to ankle stability in conjunction with their associated ligaments, the lateral ligament complex and medial/deltoid ligament respectively.

There has been considerable historical debate regarding the significance of the contribution of the medial malleolus to ankle joint stability. Yablon et al (1977) concluded that the lateral malleolus is fundamental in anatomical reduction of bimalleolar fracture patterns as the talus always faithfully followed the lateral malleolus upon reduction. [6] This was confirmed with cadaveric ankle stress testing which found that upon sectioning of the deltoid ligament or fracture of the medial malleolus ankle stability was minimally affected. Yablon's theory went against the views of others including Hughes (1980) who felt that it was the medial malleolus, which helped to re-establish a stable and congruent mortice. [7] In fact, up until this point the majority of surgeons considered the medial malleolus to be the most important stabiliser and consequently unstable bimalleolar ankle fracture dislocations were commonly treated with open reduction and internal fixation of the medial malleolus in conjunction with closed reduction of the lateral malleolus.

When assessing load bearing in the ankle joint the majority of the bodyweight is distributed over the central zone of the distal tibial plafond. During standing and walking 90% of the loading occurs in this area with the remaining load being shared between the medial and lateral malleoli. [8] Consequently good results have been published in patient cohorts with conservatively managed isolated medial malleolus fractures. Hersovici et al (2007) identified 57 patients with conservatively managed isolated medial malleolus fractures, accepting any degree of fracture reduction. [9] Only 2 cases required further treatment with an overall union rate of 96%. In general patients reported good outcomes as per the SF-36 form and AOFAS hindfoot and ankle score. Importantly there were no cases of medial instability, skin compromise, malalignment of the mortice or post-traumatic degenerative changes after a mean 3-year follow-up. They concluded that isolated medial malleolus fractures could be treated non-operatively, but consideration should be given to fixation in the cases of bimalleolar and trimalleolar fracture dislocations, which were deemed more inherently unstable.

Any operation, especially on the foot and ankle is associated with a risk of surgical site infection (SSI), particularly in elderly patients who may have contributing risk factors such as diabetes, immunosuppression and peripheral vascular disease. Infection rates between 8% and 13% have been quoted, with up to 10% requiring further surgery for removal of metalwork of wound debridement. [10,11] With this in mind, the benefits of minimising skin incisions and implantation of metalwork are clear. Over the last 12-months a number of surgeons in our trauma unit have left the medial malleolus without fixation during bimalleolar and trimalleolar fracture surgery. To our knowledge only one randomised controlled trial has been conducted on this

subject by Hoelsbrekken et al (2013) and is discussed in more detail below. [12] A further randomised controlled trial based at our trauma unit could provide additional key information that would hopefully take us closer to settling this heavily contended debate.

1.1 Current literature

Hoelsbrekken et al (2013) conducted up a prospective randomised controlled trial recruiting one hundred patients with bimalleolar and trimalleolar ankle fractures. Randomisation created two groups: fixation and non-fixation of the medial malleolus after stabilisation of the distal fibula. [12] Four patients in the non-fixation group developed radiological non-union on the medial side, but reported no functional deficits with this and comparable PROM results to the fixation group. There was a significantly lower tourniquet time/operative time in the non-fixation group (75 minutes vs. 102 minutes, $p<0.01$). More patients in the surgical group suffered a mal-union of the medial malleolus, required repeat surgery, and had subsequent radiographic osteoarthritis. This however, was not statistically significant. The authors concluded, rather conservatively and cautiously, that non-operative management was a possible treatment option but despite the advantages shown stopped short of making a clear recommendation because of their uncertainty relating the long-term consequences of medial-sided non-union.

Study	Intervention<br>No of patients	Males/Females<br>Mean age (yrs)	Patient reported outcome measures	Comments
**Hoelsbrekken (2013)**[12]<br>*PRCT*	Non-operative vs ORIF of medial malleolus<br>n=100<br>Non-op: n=45<br>Op: n=37<br>No follow-up: n=18	31/51<br>53	OMAS<br>AOFAS	All patients (≥18yrs)<br>18 lost to follow-up (100 patients initially)<br>Mean FU 44 months<br>No statistical difference in OMS + AOFAS between groups<br>Increased infection in non-fixation group, but not significant (p=0.55)<br>Four cases of medial malleolus non-union in non-op group (although not symptomatic)<br>Reduced tourniquet time in non-fixation group (p<0.01)

Table 2.1: Single previous RCT comparing fixation of the medial malleolus with non-operative treatment.[12]

2 RESEARCH AIMS

2.1 Primary aim and null hypothesis

The aim of this trial is to determine whether any difference exists in the primary outcome measure (OMAS) at one-year post injury between open reduction & internal fixation (ORIF) of associated well-reduced medial malleolus fractures AND non-operative management of medial malleolus fractures in patients undergoing surgery for an unstable fracture of the ankle.

Our primary null hypothesis is that there is no difference in outcome (primary measure – OMAS) after one year between ORIF of associated medial malleolus fractures AND non-operative management in patients undergoing surgery for an unstable fracture of the ankle.

2.2 Secondary null hypothesis

The secondary aim of this trial was to determine whether any difference exists in the complication rate at one-year post injury between ORIF of associated medial malleolus fractures AND non-operative management in patients undergoing surgery for unstable fracture of the ankle.

Our second null hypothesis is that there is no difference in outcome (secondary measure – complications) after one year between ORIF of associated medial malleolus fractures AND non-operative management in patients undergoing surgery for an unstable fracture of the ankle.

3 PATIENTS AND METHODS

3.1 Patients and centres

This trial will include adult patients (≥16 years) presenting to the single centre; Edinburgh Orthopaedic Trauma Unit, Royal Infirmary of Edinburgh, with an isolated unstable fracture of the ankle joint requiring operative intervention. The trial will commence once NHS research ethics committee (REC) approval and NHS R&D Management approval is granted.

All patients will be treated in the Emergency Department (ED) with closed reduction and casting under procedural sedation and then referred to Orthopaedics via the on-call service. Patients will be considered for the trial if the following criteria are met:

1. Aged 16 years or older.
2. An unstable fracture dislocation of the ankle joint, defined as a bimalleolar or trimalleolar fracture pattern with or without any of the following:

   - Radiographic evidence of talar shift
   - Posterior malleolar fracture of >25% articular involvement or >2mm step-off
   - Syndesmosis injury

Patients who consent to participate in the trial will be enrolled into the trial pre-operatively, but the result of their randomisation will not be revealed until after fibular fixation and assessment of medial malleolus reduction intra-operatively. Patients will

only be eligible to continue in the trial if the medial malleolus reduces to acceptable limits without open reduction (≤2mm of displacement as seen on a radiographic antero-posterior view). If the medial malleolus fracture does not reduce within this 2mm limit, the patient will require formal open reduction and internal fixation and consequently not suitable to continue as per their pre-operative randomisation. In this situation, the result of randomisation will not be revealed and the participant will be excluded from the trial at this stage.

3.2 Inclusion criteria

1. Age ≥16 years
2. Able to consent to treatment
3. Unstable fracture dislocation of the ankle joint requiring operative intervention as defined in section 3.1
4. Closed injury
5. Weber B & Weber C fractures
6. Surgery date within two weeks of date of fracture

3.3 Exclusion criteria

1. Patients unable to comply with post-operative data gathering including completing questionnaires in English language
2. Additional lower limb injury, which may impact on patient rehabilitation
3. Open fracture
4. Confirmed severe associated neurovascular injuries
5. Distal tibial intra-articular fractures/ pilon type injuries
6. Supination-adduction type 2 (SAD-2) fracture configurations with a medial malleolus vertical shear fracture
7. Patients medically unfit for surgery
8. Patients declining operative management
9. Non-residents, unable to return to the unit for follow-up for a period of 1-year
10. Current engagement in a pharmaceutical/drug trial
11. Where the treating surgeon does not feel that inclusion in the trial is in the patients' best interest either due to the fracture pattern or patient factors

3.4 Patient identification and consent

All adult patients (≥16 years) presenting with an unstable fracture dislocation of the ankle joint that satisfy the inclusion and exclusion criteria will be invited to participate in our study. All adult patients with an ankle fracture best treated operatively are eligible for enrolment in this study regardless of sex, race or ethnicity. Vulnerable

populations, including patients unable to give consent and complete post-operative questionnaires, will not be recruited.

Patients will be predominately recruited either in the Emergency Department (ED) or on the Orthopaedic ward when they are admitted pre-operatively. A small number of patients may be recruited from the outpatient clinic where a patient may initially have had a suspected stable fracture, which has then displaced on subsequent radiographs or when patients are referred into the service from other hospitals. The treating on-call clinical team will introduce appropriate patients to the study and initiate the process of informed consent. A patient information sheet will be provided for them to read before agreeing to take part ***(see attached patient information sheet).*** If the patient agrees, a member of the research team will review the study protocol in detail and address any questions the patient may have. If the patient is willing to participate, the research team member will complete the informed consent process. If this is during an out-of-hours period, consent will be taken by the appropriately qualified on-call orthopaedic trainee or consultant. All trainees and consultants within the unit will have ongoing briefing regarding the aims and methodology of the trial. With the permission of the patient, a letter will be sent to their General Practitioner informing them of their involvement in the trial ***(see attached letter to GP).***

Patients will be given a copy of their consent form ***(see attached patient consent form)***, informed that their participation is voluntary and that they can withdraw at any time during the study without detriment to their normal care in any way. Patients may take as long as they like to consider participation, provided that they still meet

all the eligibility criteria documented above. Patients who require additional time to make a decision will be contacted the following day by a further face-to-face inpatient discussion. They will also be given the contact details of an Orthopaedic specialist, independent of the trial to allow them access to further information if they require. Mr John Keating, Consultant Orthopaedic Surgeon has agreed to be an independent point of contact for the trial.

Upon agreeing to take part, patients will be randomised into one of two treatment groups intra-operatively by closed opaque envelope: operative or non-operative management of the medial malleolus fracture. Randomisation will be stratified according to age to allow an even distribution of both young (<65 years) and older (≥65 years) patients between the operative groups. The result of their randomisation will only be revealed if the fracture reduces spontaneously following lateral malleolus fixation with ≤2mm of residual displacement. On enrolment, a data collection form will be started with demographic and injury-related information collected. Regardless of the treatment randomised, the patients will be followed up at the following post-operative stages: 2 weeks, 6 weeks, 3 months, 6 months and one year. This will be either in person in the outpatient clinic or via postal questionnaire (assessment points 2.10).

3.5 Interventions

Patients randomised to operative management of the associated medial malleolus fracture will be treated routinely with standard medial malleolus fixation techniques. The most common being the use of 2x 3.5mm partially threaded cancellous screws (35mm – 45mm length) inserted at 90° to the fracture line, following satisfactory open reduction. Other techniques, used far less frequently include the use of a tension band wire construct and Kirschner wires. This is usually reserved for smaller or comminuted fragments, which would not hold a 3.5mm screw sufficiently. The technique employed will be at the discretion of the treating surgeon and aims to reproduce the decisions that are made in day-to-day trauma care. Those participants who are not suitable for randomisation intra-operatively as the medial malleolus does not reduce within acceptable limits will be excluded from the trial. The result of their randomisation will not be revealed and their envelope will be return to the study office and allocated in order to the next eligible patient. This will limit disruption to the randomisation sequence. The patient subsequent care of that patient will then be at the discretion of their treating consultant.

Post-operative immobilisation and weight bearing restrictions will be at the discretion of the treating surgeon. This is determined by a number of factors including injury/fracture pattern, bone quality, co-morbidities and patient compliance. This again reflects everyday practice when managing this common orthopaedic injury. However, the default immobilisation will be in a removable supportive orthosis (walking boot) and patients will be allowed to fully weight bear unless there is a clinical indication, as highlighted above. Post-operative physiotherapy will be

arranged at the discretion of the treating surgeon, as occurs in routine clinical practice.

4 OUTCOME AND FOLLOW-UP ASSESSMENTS

4.1 Primary outcome

Our primary outcome measure is the Olerud-Molander Ankle Score (OMAS).[13] The OMAS is a functional rating scale developed in 1984, which has been used extensively as a research tool in foot and ankle surgery. It includes nine parameters: pain, stiffness, swelling, stair climbing, running, jumping, squatting, supports and work/activities of daily living. A final score is awarded from 0 – 100, with 100 representing a better functional outcome. This score will be monitored at the set assessment points (2.10) with the one year score defined as the final primary outcome.

4.1.1 Power analysis

Prior to the study a power analysis determined the number of patients required in each arm of the trial. The primary outcome measure will be the OMAS at one year. To show a clinically meaningful difference in means OMAS at one year between the groups of 10 points, assuming a common standard deviation of 20 points, 80% power and 5% level of significance we would require 64 participants per groups (i.e. a total of 128). However, to account for potential dropouts through the duration of the study we will increase this by 20% to 77 per group (i.e. a total of 154). A p value of <0.05 will be considered statistically significant.

Statistical analysis for the trial will be performed by an independent statistician, Cat Graham, employed through the local University statistics department/Edinburgh Clinical Research Facility.

4.2 Secondary outcomes

Secondary outcome measures will include any of the following occurring during the study period:

- Complications including superficial & deep infection, nerve injury, chronic/complex regional pain syndrome, failure of fixation, re-operation, non-union and hardware complications.
- Individual components of the Olerud-Molander Ankle Score.
- Manchester-Oxford Foot Questionnaire (MOXFQ) (Isis Innovation Ltd, Oxford, UK).[14] A validated and reliable PROM for surgery of the foot and ankle, it consists of 16-items from three domains: walking/standing (7 items), pain (5 items) and social interaction (4 items). Each item is scored on a 5-point Likert scale ranging from no limitation to maximum limitation (0,1,2,3,4). A raw score out of 64 is then converted to a 0 – 100 metric score, with 100 being awarded for the most severe limitation.
- EQ-5D-5L: EQ-5D™ is a standardised instrument for use as a measure of health outcome.
- Operative tourniquet time.
- Pain assessment (visual scale 1 – 10).
- Satisfaction with service (visual scale 1 –100).
- Time to taken to return of activities of daily living (ADL).
- Time taken to return to work and sport (if relevant).
- Radiographic assessment using standard anteroposterior (AP) and lateral radiographs of the ankle joint. Outcome will also be assessed in detail with regards to fracture position on healing, radiographic complications, union,

failure of fixation and the development of radiographic degenerative changes. Pre-operative/injury radiographs will be analysed by an expert blinded to the subsequent treatment. The two weeks, 6 weeks, and one-year radiographs will be analysed but blinding to treatment method will not be possible.

4.2.1 Statistical Analysis

The primary outcome of total OMAS at 12 months will be compared between the two treatment groups using a two-sample t-test or non-parametric equivalent as appropriate. This method will also be used to compare the other continuous outcome measures between the two treatment groups i.e. MOXFQ, OMAS individual component scores etc. The pattern of change in continuous measures over the study period will be presented graphically broken down by treatment allocation. Comparison of binary outcomes such as presence of non-union, reoperation etc. will be compared between the two treatment groups using a binominal test for the comparison of proportions.

4.3 Assessment Points

All follow-up assessment will take place during follow-up visits initially with the treating consultant surgeon's team.

Follow-up assessment will be collected over a one-year period (2 weeks, 6 weeks, and one year). Routine follow-up in our institution for patients who have sustained an ankle fracture requiring operative intervention varies. Frequently outpatient clinic reviews may be conducted with radiographs at 2 weeks, 6 weeks and one year. Trial participants will not require outpatient review at 3 months and 6 months, as we do not intend to perform radiographs at this stage. Data collection on these two occasions will be performed purely through postal questionnaires. The flexibility around assessment points will be as follows: 2 weeks (+/- 3 days), 6 weeks (+/- 1 week), 3 months (+/- 2 weeks), 6 months (+/- 4 weeks), one year (+/- 6 weeks).

At each visit physical examination, treatment, complications and re-operation (e.g. hardware removal) for each patient will be recorded. Participants will be asked to complete their outcome scores independently, as they would with the postal questionnaires. This will reduce potential bias produced by the presence of a research investigator influencing patient selection. The presence or absence of a medial sided incision and obvious differences with respect to metalwork on radiographs means neither the patient nor the investigator can be blinded to the treatment group. We will also follow up patients' records to assess whether they underwent any subsequent surgery on the affected ankle during the study period. This would include debridement/irrigation for infection and/or removal of metalwork for a variety of reasons. Assessment points are found below in Table 4.1.

	Baseline/ Injury	Week 2	Week 6-8	Week 12*	Week 26*	Week 52
**Informed Consent**	X					
**Demographics**	X					
**Inclusion/Exclusion Criteria**	X					
**Randomisation**	X					
**Radiographs**	X	X	X			X
**OMAS**			X	X	X	X
**MOXFQ**			X	X	X	X
**EQ-5D-5L**			X	X	X	X
**Wound review +/- suture removal**		X	X			
**Pain score**			X	X	X	X
**Return to work & sport**		X	X	X	X	X
**Satisfaction**		X	X	X	X	X
**Complications**		X	X	X	X	X

Table 4.1: Schedule of assessments. *Data collected purely through postal questionnaire.

OMAS: Olerud-Molander Ankle Score; MOXFQ: Manchester-Oxford Foot Questionnaire, EQ-5D: EuroQol-5D

5 ETHICAL STATUS AND FUNDING

5.1 Ethical Status

This study has not yet been approved by NHS Research Ethics Committee (South East Scotland REC 02) and by NHS Lothian R&D. Application for this is in progress.

5.2 Funding

Funding will be provided by the Scottish Orthopaedic Research Trust into Trauma (SORT-IT). There will be no additional costs for the NHS.

5.3 Data Protection

All Investigators and study staff involved with this study will comply with the requirements of the Data Protection Act 1998 with regard to the collection, storage, processing and disclosure of personal information and will uphold the Act’s core principles. Access to collated participant data will be restricted to the study team. Computers used to collate the data will have limited access measures via user names and passwords. Published results will not contain any personal data that could allow identification of individual participants. Participants will be provided with a summary of the study results at their request. This will be sent via postal service.

5.4 Data Storage

All paper records with patient identifiable data will be kept in a locked cupboard in the SORT-IT office. The Chief Investigator will be responsible for the key. All computer records will have limited access via user names and passwords, and no identifiable data will leave the hospital computer system. Records will be kept for 5 years to allow follow up reviews of the accuracy of our conclusions.

5.5 Confidentiality

All evaluation forms, reports, and other records will be identified in a manner designed to maintain participant confidentiality. All records will be kept in a secure storage area with limited access. Clinical information will not be released without the written permission of the participant. The Investigator and study staff involved with this study will not disclose or use for any purpose other than performance of the study, any data, record, or other unpublished, confidential information disclosed to those individuals for the purpose of the study. Prior written agreement from the sponsor or its designee must be obtained for the disclosure of any said confidential information to other parties.

6 References

1. Court-Brown C.M., Caesar B. Epidemiology of adult fractures: A review. Injury 2006;37(8):691-7.

2. Donken CC, Al-Khateeb H, Verhofstad MH, van Laarhover CJ. Surgical versus conservative interventions of treating ankle fractures in adults. Cochrane Database Syst Rev. 2012;8;CD008470

3. Salai M, Dudkeiwicz I, Novikov I, Amit Y, Chechick A. The epidemic of ankle fractures in the elderly: Is surgical treatment warranted. Arch Orthop Trauma Surg. 2000;120:511-3.

4. Court-Brown C, McBirnie J, Wilson G. Adult ankle fractures: an increasing problem? Acta Orthop Scand 1998;69:43-7.

5. Pott P. Some few general remarks on fractures and dislocations: 175B. Clin Orthop. 2007;458:40-1.

6. Yablon IG, Heller FG, Shouse L. The key role of the lateral malleolus in displaced fractures of the ankle. J Bone Joint Surg [Am]. 1977 Mar;59(2):169-173.

7. Hughes J. The medial malleolus in ankle fractures. Orthop Clin North Am. 1980;11:649-60.

8. Kimizuka M, Kurosawa H, Fukubayashi T. Load-bearing pattern of the ankle joint: contact area and pressure distribution. Arch Orthop Trauma Surg 1980;96:45-9.

9. Herscovici D, Scaduto JM, Infante . Conservative treatment of isolated fractures of the medial malleolus. J Bone Joint Surg [Br]. 2007;89B:89-93.

10. Pagliaro AJ, Michelson JD, Mizel MS. Results of operative fixation of unstable ankle fractures in geriatric population. Foot Ankle Int. 2001;22:399-402.

11. Zaghloul A, Haddad B, Barksfield R, David B. Early complications of surgery in operative treatment of ankle fractures in those over 60 : A review of 186 cases. Injury. 2014;45:780-783.

12. Hoelsbrekken SE, Kaul-Jensen K, Morch T et al. Nonoperative treatment of the medial malleolus in bimalleolar and trimalleolar ankle fractures: A randomised controlled trial. J Orthop Trauma. 2013;27:633-637.

13. Olerud C, Molander H. A scoring scale for symptom evaluation after ankle fracture. Arch Orthop Trauma Surg. 1984;103(3):190-4.

14. Dawson J, Doll H, Coffey J, Jenkinson C. Responsiveness and minimally important change for the Manchester-Oxford foot questionnaire (MOXFQ) compared with the AOFAS and SF-36 assessments following surgery for hallux valgus. Osteoarth Cart. 2007;15:918-931.
