# Supplementary material for: Operative vs Nonoperative Management of Unstable Medial Malleolus Fractures: A Randomized Clinical Trial
Source: JAMA Netw Open. 2024 Jan 18;7(1):e2351308. doi: 10.1001/jamanetworkopen.2023.51308 (PMC10797457; doi:10.1001/jamanetworkopen.2023.51308)
Supplement: Supplement 2. — eTable. Perioperative Data for the MOON Trial eFigure 1. A Radiographic Example of a Type-C Medial Malleolar Fracture That Progressed to Satisfactory Radiographic Union eFigure 2. A Radiographic Example of a Type-B Medial Malleolar Fracture That Progressed to an Asymptomatic Radiographic Nonunion [file jamanetwopen-e2351308-s002.pdf]

Supplementary Online Content

Carter TH, Oliver WM, Bell KR, Graham C, Duckworth AD, White TO. Operative vs nonoperative management of unstable medial malleolus fractures: a randomized clinical trial. *JAMA Netw Open*. 2024;7(1):e2351308. doi:10.1001/jamanetworkopen.2023.51308

eTable. Perioperative Data for the MOON Trial

eFigure 1. A Radiographic Example of a Type-C Medial Malleolar Fracture That Progressed to Satisfactory Radiographic Union

eFigure 2. A Radiographic Example of a Type-B Medial Malleolar Fracture That Progressed to an Asymptomatic Radiographic Nonunion

This supplementary material has been provided by the authors to give readers additional information about their work.

eTable. Perioperative Data for the MOON Trial

n/% unless otherwise stated	FIXATION (n=78)	NON-FIXATION (n=76)
Median days to surgery (IQR)	2 (1-3)	2 (1-4)
Grade of surgeon		
Consultant	7 (9)	5 (7)
Trainee/fellow	71 (91)	71 (93)
Fibular fixation		
Plate and screws	56 (72)	49 (65)
Intramedullary nail	21 (27)	23 (30)
Syndesmosis screws	1 (1)	4 (5)
Medial malleolar fracture fixation		
1x screw	44 (56)	n/a
2x screw	32 (42)	1 (1)
TBW	1 (1)	n/a
Plate	1 (1)	n/a
Medial malleolar fracture reduction		
Good (anatomical)	n/a	39 (51)
Fair (≤2mm)	n/a	32 (42)
Poor (>2mm)	n/a	5 (7)
Syndesmosis fixation	16 (21)	22 (29)
Posterior malleolus fixation	6 (8)	2 (3)
Tourniquet used	73 (94)	68 (89)
Mean tourniquet time (range, SD)	77 (39-124, 18.1)	55 (30-92, 15.1)
Post-operative immobilisation		
Removable orthosis	76 (97)	71 (93)
Plaster	2 (3)	5 (7)
Post-operative weight bearing status		
Full	63 (81)	49 (64)
Partial	0	3 (4)
Non	15 (19)	24 (32)
Post-operative VTE prophylaxis prescribed	11 (14)	13 (17)

Abbreviations: IQR, interquartile range; TBW, tension band wiring; VTE, venousthromboembolism

© 2024 Carter TH et al. *JAMA Network Open.*

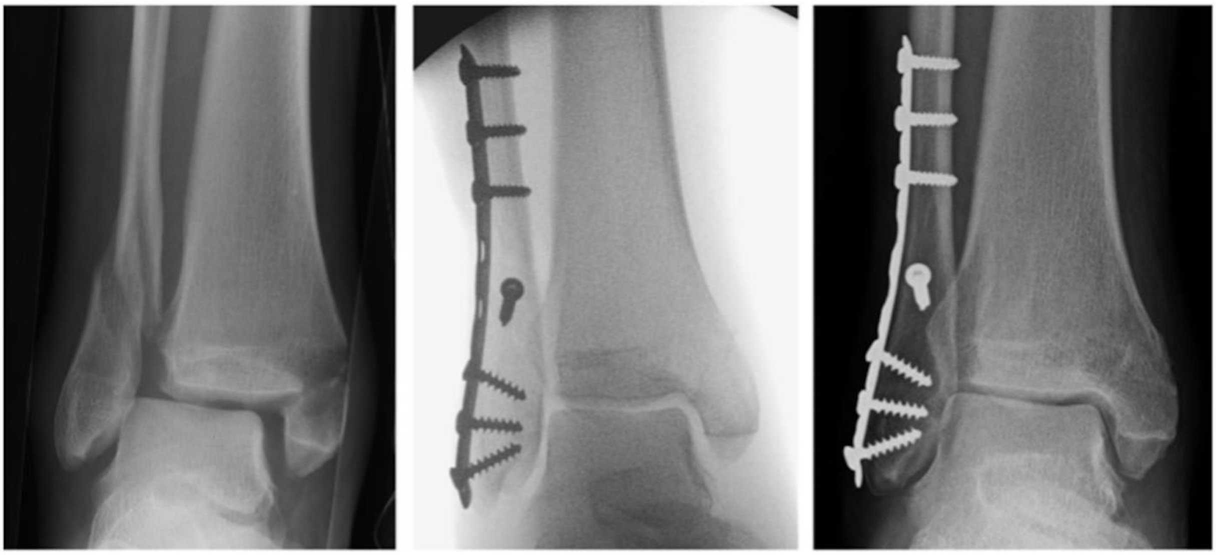

eFigure 1. A Radiographic Example of a Type-C Medial Malleolar Fracture That Progressed to Satisfactory Radiographic Union

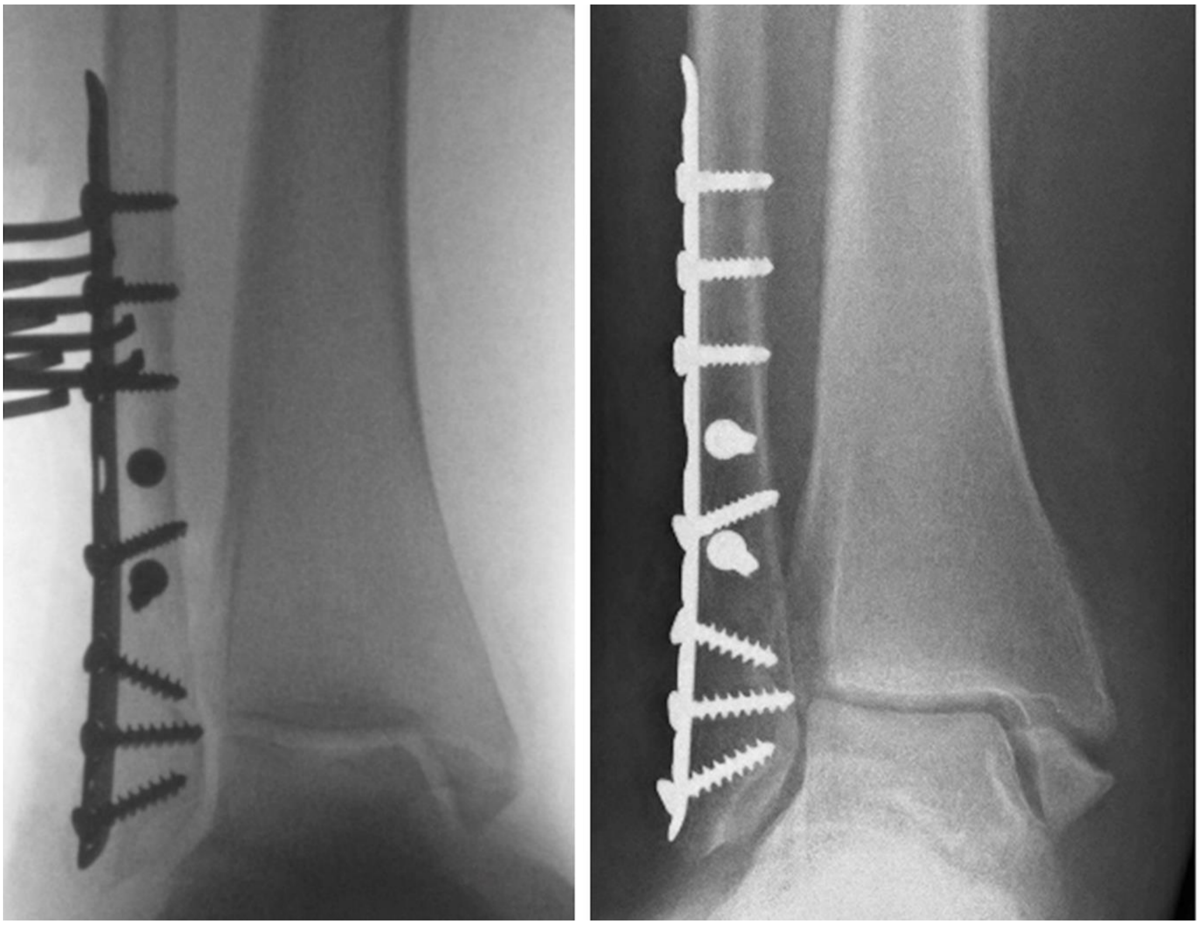

eFigure 2. A Radiographic Example of a Type-B Medial Malleolar Fracture That Progressed to an Asymptomatic Radiographic Nonunion

© 2024 Carter TH et al. *JAMA Network Open.*
