# Supplementary material for: Operative vs Nonoperative Management of Unstable Medial Malleolus Fractures: A Randomized Clinical Trial
Source: JAMA Netw Open. 2024 Jan 18;7(1):e2351308. doi: 10.1001/jamanetworkopen.2023.51308 (PMC10797457; doi:10.1001/jamanetworkopen.2023.51308)
Supplement: Supplement 3. — Data Sharing Statement [file jamanetwopen-e2351308-s003.pdf]

Data Sharing Statement

Carter. Operative vs Nonoperative Management of Unstable Medial Malleolus Fractures. *JAMA Netw Open*. Published January 18, 2024. doi:10.1001/jamanetworkopen.2023.51308

Data
Data available: Yes
Data types: Deidentified participant data
How to access data: c.graham@ed.ac.uk - trial statistician
When available: With publication

Supporting Documents
Document types: Informed consent form
How to access documents: carter.tom@doctors.org.uk
When available: With publication

Additional Information
Who can access the data: researchers whose proposed use of the data has been approved by the trial investigators
Types of analyses: for approval by the trial investigators
Mechanisms of data availability: after approval of a proposal by the trial investigators
Any additional restrictions: n/a
